# Evidence on article 5.3 of FCTC (tobacco industry interference in tobacco control activities) in India- a qualitative scoping study

**DOI:** 10.1186/s12889-021-11773-x

**Published:** 2021-10-14

**Authors:** Sonu Goel, Sitanshu Sekhar Kar, Madhur Verma, Parthibane Sivanantham, Bijay Nanda Naik, Deepti Gupta

**Affiliations:** 1 Department of Community Medicine and School of Public Health, Post Graduate Institute of Medical Education and Research, Sector 12, Chandigarh, 160012 India; 2 Public Health Masters Program at School of Medicine, University of Limerick, Limerick, Ireland; 3 Faculty of Human and Health Sciences, Swansea University, Swansea, United Kingdom; 4 Department of Preventive & Social Medicine, Jawaharlal Institute of Postgraduate Institute of Medical Education & Research, Puducherry, India; 5 Department of Community and Family Medicine, All India Institute of Medical Sciences Bathinda, Bathinda, Punjab India; 6 Community & Family Medicine, All India Institute of Medical Sciences Patna, Patna, India; 7 Department of English and Cultural Studies, Panjab University, Chandigarh, India

**Keywords:** Tobacco industry, Tobacco industry interference, FCTC, Tobacco legislations

## Abstract

**Background:**

The Tobacco Industry (henceforth TI) yearns to portray itself as being “socially responsible” and fights for the decision-making positions; that are it used to deter, delay or dilute tobacco control measures. There is little documented evidence of Tobacco Industry Interference (henceforth TII) from India, the scope of their interference and challenges faced by the experts for effective tobacco control. This research study seeks to cover this significant gap in the literature on the TI of India.

**Methods:**

A cross-sectional qualitative research design, based upon in-depth interviews (*N* = 26), was used to explore the key stakeholders’ opinions regarding TII in India. The interviews used a set of questions to collect information about the participant’s roles and responsibilities in tobacco control, the nature of TII faced by the participants, means of influence by TI, barriers and challenges to tobacco control efforts.

**Results:**

Most of the respondents were engaged in tobacco control, training, advocacy and awareness generation activities for 5–10 years or more. The respondents defined the TI and its scope as per their experience with the help of the power ranking methodology. Most of them perceived TI as ‘manufacturers’ while others consider them as ‘advertisers’, ‘public relation companies’, ‘wholesalers’, ‘vendors’, and ‘Government firms with TI stocks. The research team identified six significant domains: influencing the policy and administrative decisions, Interference in the implementation of tobacco control laws and activities, false propaganda and hiding the truth, manipulating front action groups (FAG), rampant tobacco advertising and promotion activities and others under which TII activities were classified. Most respondents believed that TI players were interfering in the policy decisions, implementing the tobacco control laws and activities and manipulating the FAG. A detailed taxonomic classification of the TII strategies that emerged from our analysis was linked to article 5.3 of FCTC.

**Conclusions:**

The study documented a significant level of TII in different domains, with stakeholders acting at various hierarchical levels. Thus providing insight into the tactics of the TI in order to enable stakeholders to anticipate and pre-empt the kinds of alliances the TI may attempt to build; stimulating academicians and researchers to undertake in-depth analysis into various strategies and therefore underscoring the need to ensuring transparency in official interaction with the TI and its representatives.

**Supplementary Information:**

The online version contains supplementary material available at 10.1186/s12889-021-11773-x.

## Background

About 275 million tobacco users reside in India [1]. This leads to the widespread production and consumption of various forms of tobacco. Due to high tobacco usage, India has one of the highest rates of oral cancer in the world, with an annual incidence as high as 10 per 100,000 among men [2]. This increased burden makes it one of the most important preventable public health problems. Therefore, the Tobacco Industry (TI) needs to be treated differently from other industries due to the fundamental conflict between their goals. Global evidence suggests that the TI is a formidable opponent of public health and development; determined to recruit new tobacco users at the cost of public health. The TI constantly portrays and reinvents itself as being “socially responsible” and therefore eligible for a seat at policy-making positions, which it uses to deter, delay or dilute tobacco control measures [3]. Public health organizations have now recognized that TI and its associated front groups and their diverse partners are the leading cause of persistent tobacco use [4]. Despite the adoption of the Cigarettes and Other Tobacco Products Act (COTPA) in 2003 and the subsequent launch of the National Tobacco Control Program in 2007, the TI of India has actively opposed and undermined the Government’s efforts to present evidence and countered evidence-backed policies that protect people from the harm of tobacco use [5, 6]. Thus, a strong political commitment at the global and regional level is needed to counter the hydra-headed TI if the health of citizens is to be protected.

Eliminating the TI from health policy design is potentially the single most effective measure that governments can adopt to protect tobacco control activities, thereby addressing the death and disease caused by the tobacco epidemic. Article 5.3 of the World Health Organization Framework Convention on Tobacco Control (WHO FCTC) and its guidelines provide a roadmap for why and how TII in health policies can be eliminated [7]. The Article calls for measures by the governments to defend health policy “from commercial and other vested interests of the TI” [8]. Also, the parties to the FCTC are obligated to objectively submit reports about the efforts made for strict implementation of the provisions, including Article 5.4, regularly to the Convention Secretariat [9]. In 2008, the Parties (COP) Conference to the FCTC adopted a series of guidelines and recommendations for the implementation of Article 5.3 [10]. WHO Article 5.3 has a set of guideline recommendations on awareness-raising (recommendations 1.1–1.2), on limiting interaction with the tobacco industry (recommendations 2.1–2.2), on the rejection of industry partnerships (recommendations 3.1–3.4), on the avoidance of conflicts of interest (recommendations 4.1–4.11), on transparency (recommendations 5.1–5.5), on the denormalization of industry “corporate social responsibility” activities (recommendations 6.1–6.4), on preferential treatment of the tobacco industry (recommendations 7.1–7.3) and state-owned tobacco industries (recommendations 8.1–8.3).

Despite global agreement through FCTC, TI has been extending its scope to control policy and legislation in high-income countries (HIC) and low and middle-income countries (LMIC) alike. Hence, the existing literature can be used to anticipate and therefore counter TII irrespective of the geographic boundaries [11, 12]. The TI has been interjecting using four main pretexts—economic activity, marketing/promotion, political activity and deceptive/manipulative activity [12]. These pretexts are implemented through a broad array of techniques, including testimonies, position papers, constituency letters contacts, and face-to-face discussions between representatives from TI and legislators for the attainment of its objective to obstruct annul, amend or halt pending legislation [13]. ﻿A detailed report of the Corporate Accountability International comprehensively captures the interference, which comprises of destabilizing and making use of legislative ambiguities, demanding a seat at Government negotiating tables, advocating voluntary regulation instead of legislation, drafting and distributing sample legislation which is advantageous to the TI, questioning and stretching government timetables for implementing laws, trying to influence legislators, gaining favour by funding government schemes on other health issues and preserving trade benefits at the cost of health [13, 14].

Besides weakening legislation in almost all developing countries that have a significant number of smokers [15–17] and launching specific scathing attacks on governments to prevent its extermination [18], TI uses tactics to undermine the importance of various scientific studies done to generate evidence regarding the harmful impact of tobacco [19]. This fact was highlighted by TI documents which clearly state that the TI prevented the diffusion of studies and innovation that can lead to more significant restrictions on smoking and therefore initiated attempts to nullify them through counter research, altering media and public opinion and lobbying administration [19–21]. Given the power and size of the TI, such repulsion can have a significant impact on legislative initiatives in different countries [22]. Also, it is now clear that geographic boundaries do not circumscribe TII and TI has adopted strategies to alter policy decisions at local levels that bring much more significant effects [23]. Reports from HICs and LMICs have explicitly delineated TII’s heinous and nefarious acts utilizing the case study approach among the stakeholders of tobacco control [13, 24–27]. However, there is little evidence from India that research has attempted to analyse the TII, its direct and indirect allies and its modus-operandi. In this context, the study’s main objective was to document the evidence on Article 5.3 of WHO FCTC implementation and related violations in India over the last decade (2007–2017). Specifically, it explored different stakeholders involved in TI, the nature of interactions between the TI and diverse actors (front groups). They influence tobacco control at the behest of the TI in India. This is among the very first attempts from India and among the few attempts globally to document the evidence of TII in tobacco control activities through evidence-based discussion [26, 27] and suggestions of the experts intended to serve as ready-made material for policymakers.

## Methods

### Study design

This is a scoping study, based upon in-depth interviews, conducted between October 2017 and March 2018. In the study, the research team intended to document the range, extent and nature of TII in India [28]. When data on the ‘perception of TI’ and ‘means of TII in India’ were obtained, they were prioritized using the power ranking method [29].

### Study settings and participants

Initially, a series of meetings among the investigators of two government institutes of national excellence of India (referred to as Institute 1 and Institute 2, respectively henceforth) along with the representatives from funding partners viz. International Union against TB and Lung Diseases (The Union), South East Asia, New Delhi, and WHO Country Office for India were conducted to frame the study protocol, in-depth interview schedule and the list of key informants for in-depth interviews through detailed deliberation.

### Inclusion criteria

Irrespective of the years of experience, the stakeholders of tobacco control in India who have served between the years 2007 and 2017 as a member of a civil society/researcher/program manager/policymaker: in various capacities (at state and national levels) and agencies (Government, private and non-governmental) were enlisted as critical informants for the study. For the power ranking method, participants were chosen depending on their expertise in tobacco control.

### Sample and sampling method

The research team approached 30 stakeholders, irrespective of the number of years of experience in tobacco control. A purposive sampling technique was employed to select the participants who could provide in-depth information on the study topic. Four respondents denied consent for the interview and we could finally interview 26 stakeholders (14 by Institute 1 and 12 by Institute 2). For employing the power ranking method, a panel of key informants who were expert in tobacco control as substantiated by the richness of information shared during in-depth interviews were selected using purposive sampling. Hence, a total of 10 members were chosen for participating in the power ranking method.

### Study tool

The in-depth semi-structured interview schedule (Supplementary File 1) was developed and used to collect the desired information. The interview schedule through a set of ten questions collected data on the socio-demographic characteristics of participant’s roles and responsibilities in tobacco control, the nature of TII faced by the participant, means of influence by TI, barriers and challenges to tobacco control efforts. The interview schedule was pre-tested and the required modifications were made after discussion. Based on the findings of in-depth interviews, ranking sheets were developed for prioritizing the ‘perceptions of tobacco industry’ and ‘means of TII in India’. (Supplementary File 2 & 3).

### Data collection

The participants were contacted over email for determining their willingness after explaining the study purpose and procedures through the consent form and Participant Information Sheet. The confidentiality and anonymity of the participants were assured. After receiving their consent, a convenient time and method for data collection (Telephone or Face-to-Face) was determined. In non-responders, two reminders (through email) within a gap of two weeks were sent, followed by prompting through telephonic reminders to boost response rates. Alongside, two researchers who had prior experience in conducting qualitative research interviews were recruited and trained in collecting data by the team of study investigators.

Each participant interview was audio-recorded and transcribed in English. During the interviews, field notes were taken to capture contextual details and non-verbal information. Field notes were shared with the participants at the end of the interviews to ascertain accuracy. Each interview lasted for approximately 40–45 min.

The power ranking method was carried out through a consultative meeting, with the results from the in-depth interviews among the investigators and selected key informants. The second investigator served as moderator for the meeting. Participants were first trained to rank responses collated from the in-depth interviews using respective questionnaires.

Rank ordering was done in two steps. In the first step, the participants were asked to rank their ‘perception of TI’ and ‘means of TII in India’ based on their priorities. In both the lists, the participants ranked the responses between 1 and 17 such that “1” represented the least representative and “17” correspond to the most representative of tobacco industry/means to TII. The individual rank orders were aggregated to develop the consolidated lists on ‘perception of TI’ and ‘means of TII in India’.

In step two, the collated lists were shown to the participants with the objective to construct the final prioritized order of responses of both lists through an iterative process. Here, the facilitator encouraged each participant to critically evaluate the aggregated lists and sought reordering of prioritized reactions if needed. When a participant suggested reordering a response in the lists, the facilitator sought a consensus from the others on the positioning and invited them to reposition it as appropriate. Adjusting the positions of responses in the lists continued until a final order of responses was agreed upon by the group members.

### Analysis

Verbatim transcripts were analyzed systematically using the inductive approach of qualitative content analysis. For analysis, each interview transcript was read several times to identify textual segments relevant to the research questions under study. Relevant texts, when identified, were initially assigned codes along with descriptive labels. Subsequently, whenever related texts were found, they were allotted to previously coined codes and newer codes for emerging textual segments. Data collection and analysis were conducted concurrently to facilitate the generation of codes according to data stemming from participant interviews.

A framework of codes developed in the process was then grouped into sub-categories, sub-themes and major themes, based on similarities and differences between the codes. Once the sub-themes and themes were generated, they were compared with the textual segments of the interview transcripts to ensure that the themes reflected the message conveyed by the participants during the interviews.

Two of the investigators were involved in data triangulation. After themes and sub-themes were generated for each transcript individually, the interview data and the field notes were exchanged electronically to validate interpretations (codes, sub-themes and themes) made. When discrepancies arose during the validation process, the interpretation by the Principal Investigator was considered to be final.

The results are presented descriptively under major themes derived from the study. They are substantiated with relevant quotations from the interview transcripts. Repeated and unnecessary words were removed from the quotes to enhance comprehension while retaining its essence.

In the power ranking method, consolidated lists of responses were developed by taking an average of ranks given by each participant to each response in the lists. In the list ‘means of TII’ (Supplementary File 3), the ‘involvement of tobacco industry role players’ across each ‘theme of TII’ was considered to be ‘present’ only when the final rank order of responses received a consensus from at least half (at least five) of the participants during the step two of the power ranking method.

### Ethics considerations

The study was approved by the Institutional Ethics Committees of Jawaharlal Institute of Postgraduate Medical Education and Research (IEC ref. no.- JIP/IEC/2017/0477 and Post Graduate Institute of Medical Education and Research, Chandigarh (IEC ref. No. PGI/IEC/2017/565). The duly signed consent forms were taken from the respondents via email before conducting the interviews, with an option to withdraw from the study at any point in time. The study was conducted and reported according to the Consolidated Criteria for Reporting Qualitative Research (COREQ) [30].

## Results

In the study, the majority of participants were men (84.6%), and most were in service (73.1%), i.e., they were serving a government-institution (38.5%), non-government institution (42.3%), or private organization (19.2%). The majority were civil society representatives (42%), and over half of them (57.7%) were engaged in tobacco control activities from the last 5–10 years. The characteristics of the study participants are summarised in Table 1.

**Table 1:** General details of the respondents invited for in-depth interviews

	Institute 1	Institute 2	Total
**Total**	**14 (100)**	**12 (100)**	**26 (100)**
**Sex**			
Male	11 (78.6)	11 (91.7)	22 (84.6)
Female	3 (21.4)	1 (8.3)	4 (15.4)
**Age**			
25-45 years	3 (21.4)	5 (41.7)	8 (30.8)
45-above	5 (35.7)	5 (41.7)	10 (38.5)
Not mentioned	6 (42.8)	2 (16.6)	8 (3.8)
**Position**			
Civil society representative	6 (42.8)	5 (41.7)	11 (42.3)
Program manger	4 (28.6)	3 (25)	7 (26.9)
Researcher	3 (21.4)	3 (25)	6 (23.1)
Policy maker	1 (7.2)	1 (8.3)	2 (7.7)
**Occupation status**			
In service	8 (57.1)	11 (91.7)	19 (73.1)
Retired	1 (7.1)	1 (8.3)	2 (7.7)
Self employed	5 (35.7)	–	5 (19.2)
**Organization**			
Government	5 (35.7)	5 (41.7)	10 (38.5)
Non-Government	4 (28.5)	7 (58.3)	11 (42.3)
Private	5 (35.7)	–	5 (19.2)
**Years of Association with tobacco control**			
5–10 years	10 (71.4)	5 (41.7)	15 (57.7)
11–20 years	2 (14.3)	5 (41.7)	7 (26.9)
20 years above	2 (14.3)	2 (16.6)	4 (15.4)

Almost all respondents perceived the tobacco industry as ‘Manufacturers’ (Mean score = 9) while more than half of them identified ‘advertisers’, ‘public relation (PR) companies’, ‘wholesalers’, ‘vendors’, and ‘Government firms having TI stocks’ as the tobacco industry. (Table 2). The power ranking method on prioritizing the players of the tobacco industry’s involvement in various themes of TI interferences showed that the ‘manufacturers’ and ‘PR companies’ were involved in all six types of industry interference pretexts identified in the study. Industry-led unions and farmer’s corporations were involved in all types of interferences except Tobacco Advertising, Promotion and Sponsorship (TAPS) activities. The industry-sponsored hospitality sector was also involved in all types of interference except hampering the implementation of tobacco control in the country **(**Table 3**)**. The majority of role players, including ‘wholesalers’, ‘bidi rollers’, ‘politicians’, ‘bureaucrats’ and ‘civil society organizations’, were associated with four types of industry-led interferences in the country. **(**Table 4**)**

*“TI are various kinds of people who lead or who are part of tobacco production and selling (tobacco). Whosoever works for furthering the interests of TI are also part of TI; they may be peers, packagers, marketers, or advertisers are also TI.” (One respondent).*

*“It's an established industry which is present in both organized and unorganized industry and it is well connected (politically).” (a health professional)*

**Table 2:** Perception of study participants regarding Tobacco Industry using ‘Power Ranking Methodology’

Stakeholders	Average score (***N*** = 10)
Manufacturer	9
Wholesaler	6.8
Vendors	6.2
Advertisers	7
PR Company	6.7
Government with tobacco stocks	5.8
Government without tobacco stocks	2.2
Tobacco union workers	4.6
Farmers	3.8
Farmers corporations	4
Pension funds and other Financial incentive schemes	2.6
Banks and financial institutions	3.6
Bidi rollers	4.6
Politicians	5
Bureaucrats	4
Civil Society Organization	3.6
Hospitality Industry	0.6

**Table 3:** Role of tobacco industry in interfering tobacco control activities in India

Themes	Sub-themes
**Influencing the policy and administrative decisions**	Projected revenue generation and livelihood creation
	Providing sponsorships for government events & political parties
	Using policy loopholes
	Offering undue favors
	Lack of prioritization towards tobacco control
**Interference with implementation of tobacco control laws and activities**	Interference in the judiciary system
	Interfering with work of tobacco control officials
	Interference in functioning of NGOs who work on tobacco control
	Prompting sellers for non-cooperation with tobacco control officials
**False propaganda and hiding the truth**	Exaggerating the economic impact of tobacco and loss of livelihood
	Hiding facts about tobacco harms
	Promoting CSR activities to gain social respectability
	Hiding involvement in sponsored events
	Misguiding tobacco growers
**Manipulating through front action groups**	Instigating protests and non-cooperation
	Threatening tobacco shop owners
**Rampant tobacco advertising and promotion activities**	Promoting surrogate advertisements
	Creating new customer base with attractive offers
	Support and bonus for tobacco shops and vendors
**Others**	Evidence of Government honoring tobacco industry officials
	Poor awareness on harmful effects of tobacco among ancillary stakeholders of tobacco industry
	Lack of cohesion at institutional levels for tobacco control discussion at state and national level
	Lack of understanding and support for tobacco control initiatives
	Situational priority i.e. tobacco control taking backseat over other priority issues

**Table 4:** Perception of study participants regarding involvement of “Tobacco industry role players” in various identified themes

Tobacco industry role players	Theme 1 (*Influencing the policy and administrative decision*)	Theme 2 (*Interference with implementation of tobacco control laws and activities*)	Theme 3 (*False propaganda and hiding the truth*)	Theme 4 (*Manipulating front action groups*)	Theme 5 (*Rampant TAPS activities*)	Theme 6 *(Others)*
**Manufacturer**	Y	Y	Y	Y	Y	Y
**Wholesaler**		Y	Y	Y	Y	
**Vendors**	Y	Y			Y	
**Advertisers**		Y	Y		Y	
**PR Companies**	Y	Y	Y	Y	Y	Y
**Government with tobacco stocks**	Y	Y		Y		Y
**Government without tobacco stocks**		Y		Y		Y
**Tobacco union workers**	Y	Y	Y	Y		Y
**Farmers**			Y	Y		Y
**Farmers corporations**	Y	Y	Y	Y		Y
**Pension funds and other financial incentives schemes**			Y	Y		
**Banks etc.**	Y	Y		Y		
**Bidi rollers**	Y	Y		Y		Y
**Politicians**	Y	Y		Y	Y	
**Bureaucrats**	Y	Y	Y	Y		
**Civil Society Organizations**	Y		Y	Y	Y	
**Hospitality Industry**	Y		Y	Y	Y	Y

### Interference of tobacco industry with tobacco control activities

**Fig. 1:**
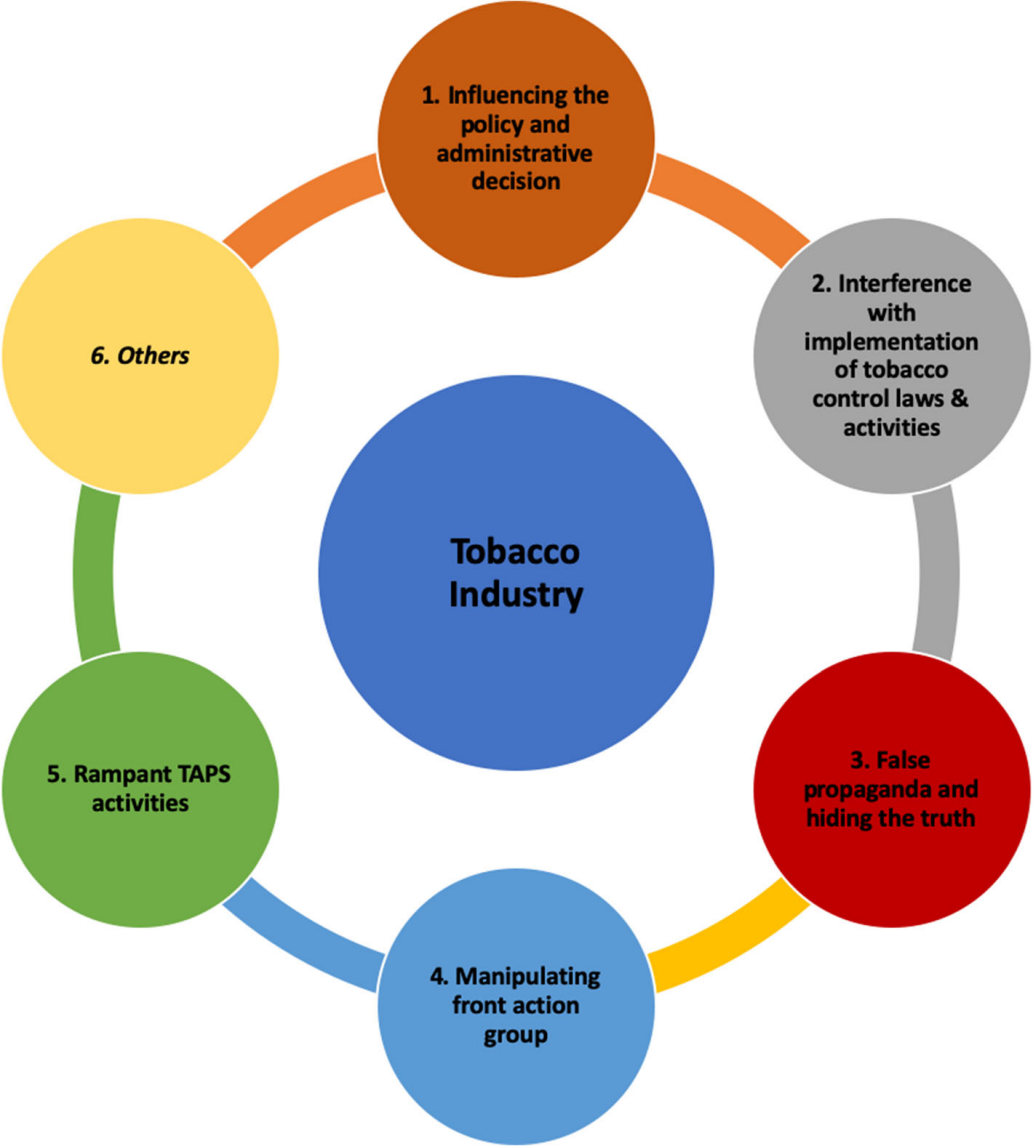
Thematic diagram showing various tactics used by tobacco industry for survival

The analysis of all the key informant interviews regarding interference of TI with tobacco control activities generated six themes and categories, as shown in Table 3 and Fig. 1.

#### Influencing the policy and administrative decisions

Participants see TI as a prime intruder in policy-making and key administrative decisions of the Government. The participants opined that TI uses monetary and power tactics to influence policymakers in placing tobacco control in the backseat.

Nearly everybody believed that TI structures significant supporters towards income for the administration and making business for some families like tobacco producers and tobacco vendors. A participant surmised that TI had been traditionally involved in various governmental development projects, which directly contradicts Article 5.3 of FCTC.

*“TI are spending crores of money in various states. They are sponsoring a lot of money in the food industry.” (A tobacco control advocate)*

*“Recently we had got a governmental order issued for increased taxation on tobacco products which was immediately reversed by our Government, possibly due to pressure (of TI). Similarly, political leaders opposed the ban of Gutka (oral chewable tobacco) in the parliament on the pretext of loss of income of farmers” (A tobacco control advocate)*

The participants felt that TI creates an impression of ‘no immediate threat associated with tobacco’ among the public, which results in a lack of public pressure on policymakers and politicians in framing effective tobacco control policies.

*“Unlike accidents and epidemics, tobacco use is not seen as an immediate danger to politicians and public, which is effectively utilized by TI. Whenever we go to public for a raid (COTPA enforcement), they question, 'why don't you close the tobacco companies rather than making the people suffer?” (A government official)*

The respondents also shared the information that many political parties and officials in the Government receive favors from TI, which undermine tobacco-related policy decisions.

*“In one of our state’s budgets, the then minister declared a 5% VAT on bidis, which was immediately reversed to zero percent on the behest of TI, which we could be due to interference from the TI.” (A government official)*

Tobacco is still not considered an illegal product and no license is needed for processing, manufacturing or selling tobacco products, unlike liquor by the law of the land. TI uses this argument to convince policymakers to make tobacco products easily accessible at various Point of Sales.

*“The biggest challenge for us is that we are one of the largest tobacco-growing and exporting countries. It's not an illegal product. TI use this argument for its easy accessibility in markets” (A health professional)*

TI takes advantage of slow legal proceedings and poor implementation of tobacco control laws. The conflicting orders from different ministries provide the opportunity for the TI to carry out promotional activities.

*“TI has people who find loopholes in government policie, which leads to slowing of legal proceedings.” (A government official)*

The participants reported undue favours being offered by the higher officials of the Government from the TI representatives in the form of gift hampers or financial support to the organizations, as a tool to influence them to take policy decisions at the behest of TI.

*“One of the Corporation officials daughter’s marriage was completely sponsored by a Gutka industry, starting from booking marriage hall to buying jewelry which was almost everything for the wedding.” (A tobacco control activist)*

Most of the respondents felt that the TI uses existing corruption within the system and week political setup to their benefit.

*“TI tries to build pressure through their political representatives to delay or dilute the policies.” (A participant).*

*“Vested interests of politicians results in lack of their will to combat Tobacco menace.” (A participant)*

Few of the respondents threw light on how TI manipulates policy making and implementation by challenging such procedures in the court by using fake documents and poorly generated scientific evidence. Respondents reported witnessing instances when the TI used artificial self-sponsored researches in its favour.

*“Smokeless tobacco lobby always tries to threaten them (Government officials). They also support high-level officials to refine the fake evidence which are provided by TI in support of their business.” (A respondent)*

#### Interference with the implementation of tobacco control laws and activities

The participants believed that TI is a well-established sector with a tremendous amount of money. They interfere with implementing tobacco control laws and activities by influencing the judiciary, implementing officials, non-government officials and merchant association or tobacco sellers. The respondents stated that legal challenges (litigations, RTIs etc.), offering undue favors, issuing threats are being used as weapons by the TI to interfere in the implementation of tobacco control laws.

*“TI has top level lawyers who challenge every favorable decision on tobacco control in the court of law, to dilute its effective implementation” (A tobacco control advocate).*

“*After a state high court had given an order about the 85% pictorial warning on tobacco packets, TI went to court several times which delayed its implementation.” (An academician and researcher)*

*“TI representatives met me with an only agenda to stop supporting govt. initiatives in tobacco control program.” (A public health activist)*

*“TI threatened me for implementing tobacco control activities in my state and even filed many RTIs to build the pressure.” (A government official)*

*“Sometimes, when we are creating awareness among shopkeepers, some agents used to come from a particular company, and question us why are we stopping their business?” (A tobacco cessation expert)*

*“Foundation for Smoke Free World associated with a multinational tobacco company, is trying to influence people who are working for tobacco control. This foundation approached many tobacco control advocates through in-person meetings and mails by presenting favours. The foundation approached President of an association but he denied their proposal. I sense that TI has used its financial muscle power to cancel FCRA licence of many associations.” (A respondent)*

Most of the respondents said that the TI creates a non-cooperative attitude among the distributors and point-of-sale owners by providing various incentives like installing and replacing advertisement boards removed during enforcement drives or compensating for losses in case of seizers or challans.

*“A point-of-sale owner told me that TI people came to him to reimburse the challan (fine) and even replaced his advertisement boards that were removed during the enforcement drives”* (An academician and researcher)

*“TI instructs the sellers of cool lips (a flavored form of smokeless tobacco) to distribute among the children for which they paid incentives.” (A tobacco control enforcing official)*

Most of the stakeholders opined that there is a shortage of awareness at all levels, i.e. among the stakeholders, the enforcers, the policymakers and the masses, which hinders the proper implementation of the tobacco control laws.

*“Lack of awareness and passion for tobacco control is the root cause of the challenges that came up during the implementation of tobacco control policies in the state.” (A Deputy Director working in tobacco control)*

#### False propaganda and hiding of truth

Responses from the interviews provided an insight into how the TI paints a bright picture in front of the public and policymakers and hide their vested interests under the garb of health promotion and social activities performed by them.

Many responses from the interviewees highlighted the fact that TI undertakes corporate social responsibility (CSR) by which they depict their companies as ethically correct and economically productive to society.

*“TI offered to support our school intervention program, and we denied it” (A tobacco control expert)*

*“Earlier, we (in NGO) used to carry out tobacco control activities in collaboration with other NGOs. But, when the tobacco companies started CSR activities, they started funding many NGOs, which resulted in rifts between NGOs to garner funds from them (TI).” (A leading tobacco control advocate)*

By discrediting scientific evidence, TI misguides the existing and future customers and discourages tobacco users from quitting.

*“They (TI) are advocating that the tobacco is not harmful, tobacco is not causing cancer. They have many such studies (false evidence) to prove their statements”. (An academician and researcher)*

Some respondents reported misleading claims by TI representatives of being a support for global tobacco regulation that aligns with the FCTC

*“A person who worked for tobacco control, later joined as the head of ‘Foundation for Smoke Free World’, an initiative by a leading multinational tobacco company, wrote a mail to about 300 people all over the globe to join their organization and the people who did not know much about TI easily accepted his proposal.” (A Behavioral Scientist)*

Tobacco growing is the only means of earning for a section of people. The TI doesn’t want the tobacco growers to switch to other means of livelihood.

*“Most of them, either tobacco growers, or tobacco users are not aware of the big industry involved. Only middlemen are aware of the industry nexus.” (A leading health professional)*

#### Manipulating front action groups

Most of the respondents stressed the manipulative powers of the TI. According to them, the main tactics used by the TI were threats and manoeuvring acts.

Threats of legal action are the popular means used by the TI to intimidate governments and activists who introduce and support effective tobacco control policies. Others provide physical threats to them. As per the respondents, the mere threat of such litigation and physical threat discourages the whole implementation process.

*“Smokeless tobacco lobby always tries to threaten our staff” (An Executive Director of an NGO)*

*“TI tries their best efforts to influence public health policies by providing them with policy loopholes and manipulating the facts. They are all directed towards the single motive of increasing their business*.*” (A Manager working with an NGO)*

*“When we asked them (tobacco sellers), why they were again selling (tobacco), they said that it was because of physical threat (from tobacco dealers)” (A tobacco cessation advocate)*

With a vested interest, the tobacco company instigates the front action groups to protest tobacco control laws citing livelihood issue and harassment.

*“I’m working closely with the government tobacco control cell. Recently, they have instigated the retailers to protest, over the livelihood issue.” (A leading health professional working for an NGO)*

#### Rampant TAPS activities

As per the respondents, the TI uses tobacco advertising, promotion and sponsorship (TAPS) to increase the consumption of their products. The TI uses deceptive, misleading, and predatory tactics to make tobacco use appear glamorous and socially acceptable while minimizing perceptions of these products’ adverse health effects.

A respondent working in the implementation sector said that the TI supports distributors and wholesalers by performing various activities like paying daily wages to petty sellers and providing signages and other posters mandated under law to these retailer/vendors etc. Similarly, by endorsing other brands and partnering with other organization for sponsored events, they indirectly advertise their company. This dissuades the existing customers from quitting tobacco use.

*“They do things indirectly, and it is like hide and seek game with them (tobacco companies); because, surrogate advertisements are still there, which we (government) need to find or somebody has to inform us.” (A public health professional)*

*“We have also taken efforts to stop various competitions sponsored by tobacco industry like 'Sun-feast competition', 'Spell bee', and 'Mangal deep' singing competitions. These are against that order (Order 242).” (An advocate)*

Besides, TI uses different tactics to create a new customer base and maintain the existing one. Lowering of price and selling components separately is another tactic.

*“We got to know that the TI came up with two cigarette contained pack. So, that it is a pack (tobacco pack) and not a loose cigarette.” (An academician)*

*“We convinced the new government to ban Gutkha. But, since then, Gutkha is being marketed with tobacco and betel leaves separately in smaller packets and in reduced rates.” (An academician).*

Flavoured chewable tobacco products at point-of-sale attract new customers, especially students and children.

*“We had recently faced difficulty to seize a product named ‘Cool lips’. This is a chewable form of tobacco sold like chocolates targeting the school children. The pictures of the product are also available on the internet.” (A tobacco control enforcing official).*

TI provides financial support in reimbursement for the penalty imposed by officials and bonuses for selling tobacco products. This encourages the tobacco sellers and vendors to continue promoting tobacco products despite legal actions.

*“Cigarette companies and dealers used to promote their products by giving some bonus or cash prizes to the shopkeepers; they (shopkeepers) want that also.” (A tobacco cessation advocate)*

#### Others

Specific themes that were generated but couldn’t be categorized into any of the above-mentioned major domains were compiled to constitute the sixth domain.

- *Government honoring TI officials (*e.g.*, a leading cigarette manufacturers CEO was awarded India’s highest civilian award):* Honoring TI officials acts as a catalyst for TI to expand its business.
- *Poor awareness of harmful effects of tobacco among ancillary stakeholders of TI (*e.g.*, tobacco plant growers, tobacco sellers)*: The TI takes advantage of the lack of awareness of harmful effects of tobacco among lower-level stakeholders.
- *Lack of common platform for tobacco control discussion (reason for ineffective, uncoordinated decision making and poor implementation of tobacco control laws)*
- *Lack of understanding of tobacco control and limited support among government officials and policymakers (especially elected politicians) concerning tobacco control.* Situational priority for other communicable diseases, inadequate funding, and lack of understanding of tobacco control among government officials, poor coordination between different government departments prompt TI to flourish.
- *Situational priority (several immediate issues are prioritized over tobacco control at the grassroots)*

### Challenges faced and the factors pertaining to the same

Many factors pose challenges at various level of tobacco control. Our respondents enumerated a list of challenges faced by them during the implementation of the tobacco-control activities. The challenges are at different levels and encroach the domain identified earlier in Table 3. Various stakeholders attempt interference in the different domains, as summarised in Table 4. The TI deploys different strategies of interference to persuade the public and decision-makers. The detailed taxonomy for these strategies has been explicitly described in Table 5 for the specific clause and recommendation of article 5.3 that is either violated or in an apparent contradiction to Article 5.3 specifically.

**Table 5:** Tobacco industry interference and actors which challenge tobacco control efforts, based on responses of tobacco control experts who participated in the study

Tactic	Goal	Key stakeholders for tobacco industry	Example	Violation or contradiction to the guiding principles of article 5.3
**Intimidation**	Use its power to harass and threaten tobacco control community	Politician and bureaucrats	Recent cancellation of permissions of tobacco control organisations	Clause 3, 9
**Creating alliances and front groups**	Present exaggerated and widespread negative impact tobacco control legislation/ tobacco control policies	Civil society, farmers networks etc.	FAIFA, State level farmers associations All India Bidi Federation	Clause 19 Recommendation 1.2
**Supporting Government agencies that assist tobacco sector**	Garner support from within the government	Ministries of Finance, Labour, Agriculture, Commerce, Tobacco Board	Various policy measures	Clause 3, 13,20,21 Recommendation 2.1, 3.1, 7.1
**Political funding**	Contribute to local and national political parties for campaign and seek favours from elected politicians	Political parties and party leaders	ITC annual reports Report of various tracts through which political funding is routed	Clause 22–23, 26–27 Recommendation 4.10, 4.11,6.4
**Lobbying**	Influence political and decision-making processes by presenting specious data and distracting officials from tobacco control	Front groups, PR firms	Lobbying by PR firms during Committee of Subordinate Legislation on pack warning (2015–16), TII	Clause 24–25 Recommendation 5.2
**Litigation**	Challenge laws and intimidate tobacco control advocates	Vendors, farmer associations, vested interest groups, Industry	Ghodawat pan masala pvt. ltd v/s the state of Maharashtra and others (WP no. 1632 OR 2012);	Recommendation 6.
**Public relations**	Shape public opinion by using media to and promote positions favourable to the tobacco industry and its allies	Media houses, PR companies, Government flagship programmes	Participation of tobacco companies in Government flagship programmes as a part of CSR	Clause 26–27 Recommendation 6.1,6.2
**Philanthropy and Corporate Social Responsibility**	Re-normalise and re-legitimize tobacco with society; gain social respectability by participating in social and economically relevant issues.	Through reputed NGOs and government schemes	Support neglected areas of investment. Eg: GPI supports vendors in flood affected areas by creating tobacco vends Partner with Govt in its flagship schemes like Swachh Bharat Abhiyan etc., floods/drought situation/ pandemic	Clause 26–27 Recommendation 6.2
**Participate in decision making with government**	Promise investments to state government	Industry associations and government bodies	Investments promised by ITC, GPI, and DS Group to states	Clause 28–29 Recommendation 7.1,7.2,7.3

#### Tobacco control not given highest priority by government officials

1. *Tobacco control is not high on the Government’s priority list, especially for departments other than health. Revenue earned from TI is projected, and the loss due to tobacco-related diseases is undermined. Limited dedicated funds or workforce is allotted for tobacco control. Trained officials are frequently transferred, which leads to the intermittent implementation of tobacco control laws.*
2. *Inadequate political support against TI and limited documented evidence*

I. **Lack of cohesion at institutional levels:** *There is no common platform for discussion and adopting strategies for tobacco control. Many departments are not aware of their roles and responsibilities.*
II. **Lack of funding from international donors and local corporates for tobacco control:** *There are not many donors for tobacco control. The corporate world seems to restrain itself from investment in tobacco control.*

#### Difficulty in implementation and enforcement

i. Lack of awareness: *Second round of the Global Adult Tobacco Survey (GATS)-India (2016–17)* [31] *has depicted adequately high awareness regarding the harmful impact of tobacco use. However, some of the enforcing officials (police), politicians and judiciary system are still unaware of the impact of tobacco use and its consequences on society. TI sponsored competitions, surrogate advertisements, and partnerships are evidence of low awareness among various stakeholders.*
ii. Differential priority and lack of interest among enforcement officials:

*Most of the enforcing officials are multitasked. For instance, they are delegated responsibly to mitigate seasonal communicable diseases (like Dengue), which poses an immediate threat. Many senior officials are not convinced enough to attend all the tobacco-related meetings.*

### Mode of approach by the tobacco industry

TI contacts different tobacco control activists and officials for preventing tampering with their business. Half of the participants reported being approached by TI in various ways. Two of the participants were approached directly, whereas three of them indirectly. One participant reported being approached by both means. Multiple reasons cited were 1) not to create awareness on harmful effects of tobacco 2) not to harass shopkeepers in the name of COTPA violations 3) enquire about tobacco cessation activities implemented by the Government. Two participants reported being threatened directly by TI for interfering with their business. A participant was offered undue favours to maintain a distance from TI’s business. Three participants reported being aware of tobacco control advocates threatened by TI.

## Discussion

The failure of the Government to adopt measures that are proven to reduce tobacco consumption is mainly due to interference in the Government’s policy-making process by the TI. This has been documented for many HICs and LMICs [32, 33] but has not yet been attempted from India, one of the major business hubs for TI. ﻿ This is the first of its kind study from India to document TII and its prevalent types. The findings can be used to construct a model applicable on a larger scale for governments of various countries to recognize and prevent TII in policy-making decisions. Constructivist grounded theory was used to identify techniques and arguments, which were later categorized into general strategies’ and finally, a taxonomy and TII activity model was created. We were able to identify six major domains under which TII activities can be classified through our qualitative analysis.

Most of the respondents agreed that TI is constantly influencing policy and administrative decisions. WHO FCTC’s Article 5.3 and guidelines took cognizance of this conflict and advocated steps to check the influence of TI on public health policy-making [9, 34]. However, the findings of a contemporary report by FCTC signals the requirement of further advancement to address this issue [35].. Like our study, a report from three South-East Asian countries has demonstrated that the TI in Thailand tries to undermine the progress made in tobacco control. By contrast, in Indonesia, it has a free hand to influence the Government. Myanmar is encouraging foreign investments, including the TI; hence, it is open to the TI despite the tobacco-control endeavor taken by the Ministry of Health and Sport [3]. Tobacco and tobacco products generate large volumes of the Indian Government’s tax revenue, which can be as high as Rs. 43,000 crores annually [36]. Even though tobacco taxation is the most economical way to control tobacco use, it is the least utilized policy measure [37]. Such a large chunk of revenue puts them in a better position to be listened to and participate in decision-making processes. TI portrays themselves as playing a major role in the economic overhauling of their region from which they are operating through revenue generation and livelihood creation, thus diverting attention from other pertinent issues [11]. Another study from the United States has also stated that the economy of the six south-eastern states- also known as *the “tobacco bloc” of the south-eastern United States*- is extremely dependent on the growing and manufacturing of tobacco. However, the jobs related to the main tobacco sector in these states is merely 1.6% [38]. Similarly, corporates sponsoring the events and organizations is a well-known tool for marketing, which corroborates the findings from the present study [35]. As per marketing literature, funding boosts the image of a corporation, connects the funding company’s name with interests significant to a specific target group, provide fruitful exposure to the product, target particular populations comprising groups that are hard to reach by conventional advertising modes and bestows fame to the company with the help of highly visible activities [39].

The results of the study show that the TI takes benefit of the policy loopholes and tries to evade the obligations**.** Previous literature has also highlighted that whenever a new law is made, or the previous one is amended, there are always certain weak points left that are mostly due to professional lobbying by the TI, who then use those loopholes for their profit [21, 40, 41]. Previous literature has already documented that TI gives undue favors to political parties in power or even in opposition to delay implementation of the legislative procedures [42, 43]. This can be better explained by a case study from Malaysia, where the Government, along with the enactment of Control of Tobacco Product (Amendment) Regulations (CTPR) 2008, initiated pictorial health warnings (PHWs). Lack of a specification for minimum permitted pack size for PHWs was a major weakness in it, which resulted in the import of cigarette packs named ‘*Sampoerna Avolution’* in the shape of small ‘lipstick’ boxes from Indonesia. There was serious manipulation in PHW on the package and it was nearly unidentifiable [44].

On the same lines, most of the stakeholders observed interference with implementing tobacco control laws and activities; which is regarded as another common form of TII tactics. One of the vital tools for improving tobacco control is litigation and judicial interpretations, which have played a beneficial role by institutionalizing laws for tobacco control in many jurisdictions across the globe [45]. However, it also remains one of the most significant challenges for the governments to protect tobacco control efforts from litigation by the TI [46]. The TI interferes with the judiciary system and tobacco control activists to delay decision-making and implementation of the rules [47]. Previous case reports have demonstrated how the threat of a potential international lawsuit can create a chilling effect by helping delay the implementation of public health policies [48]. Our respondent agreed that the lack of sustained funding to drive the tobacco control efforts had been a constant bottleneck. TI mediates this shortage by lobbying against the tobacco cessation programs, similar to observations made in other countries [49]. Tobacco control activity and effectiveness are left vulnerable if its funding is dependent on short-term funding pledges. All countries, even the poorest, have the means to reduce tobacco use if tobacco taxes are increased and funds are applied to fund comprehensive tobacco control programmes [50].

Governments in various countries have been sued by the TI to check the laws thoroughly or hamper their implementation [51–54]. In Brazil, TI brought a judicial claim arguing that the National Health Surveillance Agency (ANVISA) didn’t have legal authority for regulation of tobacco products, which resulted in a delay of six years in implementing the ban on flavours and additives in tobacco products. Similarly, in Bangladesh, due to the Bangladesh Cigarette Manufacturers’ Association’s (BMCA) interference, temporary permission to print pictorial health warnings (PHWs) on the lower parts of the pack was granted to TI by the law ministry on March 16, 2016 [55]. In Nepal, the tobacco control advocates effectively utilized domestic litigations, advocacy with politicians, legislators and other stakeholders for thwarting the TII in the enactment of comprehensive tobacco control policy in the country [56, 57]. Case reports from other LMIC suggest that constant engagement by the tobacco control advocates with the support of international tobacco control agencies and local health groups is required to counter the TII during the implementation phases of tobacco control policies national/sub-national level [53, 58–61].

As the evidence for the lethal impact of tobacco products started mounting, the companies created uncertainty and dispute regarding health risks while putting filters on cigarettes and promised research into the health effects of smoking to address growing public concern. Further, there is strong evidence from the Master Settlement Agreement in 1998, which shows that the radioactivity of tobacco smoke and its potential to cause cancer were known to TI, but they purposefully concealed this information [62]. In another instance in the USA, a network of consultants was developed by TI to advocate ventilation as a “remedy” to second-hand smoke (SHS) [63]. The sheer bulk of tobacco advertisement contributes to the erroneous notion of normalizing smoking in various contexts [64–67]. Under so-called corporate social responsibility (CSR) activities, millions of dollars are spent by TI each year, by giving scholarships to economically weaker students, funding projects for poverty alleviation, providing assistance during natural calamities; just to divert users’ concerns and maintain a good image before them [68, 69].

The industry has also constantly manipulated the front action groups [70–72]. TI coherently works with several business allies and third parties to impede effective tobacco control legislation and programmes. For instance, the International Tobacco Growers’ Association - a TI funded tobacco farmer’s lobby group - worked as a front-group in developing countries. Tobacco manufacturers have repeatedly instigated the farmers to represent their views, however, but always had a blind eye towards the long-pending concerns of the farmers, who were not being benefitted from the profit that was being generated [33]. In another instance, the TI documents state that a multinational tobacco company contributed financially to existing hospitality associations. It made its own “association” to halt the accretions in developing smoke-free environments [56, 73].

TI circumvents laws to promote their products by using innovative and sometimes covert marketing strategies despite TAPS prohibition laws [74–76]. When pressure on TI increased, indirect or proxy tobacco advertising such as dark advertising, brand stretching, corporate social responsibility (CSR) activities, promotion through films and new media such as the internet, discounts or free-gift offers, distribution of free samples, sale of tobacco products in the form of children’s sweets/toys, etc. gained impetus. Guidelines for implementing Article 13 of FCTC describe comprehensive TAPS ban to apply to all forms of commercial communication, recommendation or action and all forms of contribution to any event, activity or individual with the aim, effect or likely effect of promoting a tobacco product or tobacco use either directly or indirectly. A literature review has depicted that TI and their allies in Government were also able to use trade concerns over tobacco to successfully help prevent the implementation of TAPS for a significant durations of time [61].

This study has specific strengths. First, it is the first study from India that attempts to document and categorize the TII in preventing the implementation of effective anti-tobacco laws in the country. Second, it reports interviews from a wide range of players working in and around the TI in every sector and each part of the country for a comprehensive picture of TI. However, there are certain limitations. Even though a variety of stakeholders was interviewed, the sample size was small. Also, they were purposively selected from the existing knowledge of the investigation team. It is worth documenting here that the group of investigators were experts working at the country and state level in tobacco control who are pretty knowledgeable about the sample panel for the study. Further, there were considerable similarities and compatibility in the views of the stakeholders from varied branches and different parts within the country, indicating that they are indeed representative of the stakeholders.

There are several policy implications of this study. One, this work has highlighted various strategies traditionally used by the TI for diluting and deterring specific policies, that can further provide insights to policymakers about their nefarious tactics. Two, the listed strategies will enable stakeholders to foresee and prevent the types of alliances the TI may attempt to build. Three, this study will stimulate academicians and researchers to go in-depth into various strategies highlighted in the paper for getting more evidence on this important subject. They can also use the study to produce and communicate information to the policymakers, paying attention to the audience and language. Four, the work underscores the need for ensuring clarity in official interaction with the TI and lobbyists. Ultimately, the strategies can provide public health advocates and policymakers with a dominating position from which they can plan narratives and strategies proactively and not merely react to those of the TI. Further empirical work is required to document the country-based examples of TII and categorize them under each strategy domain presented in this study to further advance the Indian stance in countering TII.

## Conclusions

The present qualitative analysis documents the significant interference of TI in different domains with stakeholders acting at various hierarchical levels. This study can be beneficial for the public health community and policymakers at the national, regional and international levels to provide insight into the nefarious tactics of TI, enable stakeholders to anticipate and pre-empt the kinds of alliances the TI may attempt to build and stimulate academicians and researchers to undertake in-depth analysis into various strategies highlighted in the paper. The research also underscores the need for ensuring thorough transparency in official interaction with the TI and lobbyists. Future studies should generate more evidence of TII in different domains that emerged from the current paper-like tobacco pack health warnings, TAPS, Electronic Nicotine Delivery Systems (ENDS) (e-cigarettes, and so on. Suppose the public health experts or policymakers intend to face the TI for legislative enforcement. In that case, they need to choose a scientific approach by generating enough evidence against TI that exposes its true face on both the global and local front.

## Additional files

**Additional file 1.** Supplementary File 1: In-depth interview Schedule.

**Additional file 2.** Supplementary File 2: Ranking Sheet: Perception of study participants regarding Tobacco Industry using ‘Power Ranking Methodology’.

**Additional file 3.** Supplementary File 3: Questionnaire: Perception of study participants regarding involvement of “Tobacco industry role players” in various identified themes. Instruction: Prioritize (1 to 17) the role players of tobacco industry under each theme of industry’s interferences. Rank the role players from 1 to 17 such that 1 represents least involvement and 17 correspond to most involvement under a particular theme of TII.

## Data Availability

The transcripts of the in-depth Interviews analyzed during the current study are available from the corresponding author on reasonable request to protect the anonymity of the participants.

## Abbreviations

TI: Tobacco Industry
TII: Tobacco Industry Interference
WHO FCTC: World Health Organization Framework Convention on Tobacco Control
COREQ: Consolidated Criteria for Reporting Qualitative Research
TAPS: Tobacco Advertising, Promotion, and Sponsorship activities
*COTPA*: Cigarettes and Other Tobacco Products Act
*GATS*: *Global Adult Tobacco Survey*
CTPR: Control of Tobacco Product (Amendment) Regulations 2008
PHW: Pictorial health warnings
