# Supplementary material for: Evidence on article 5.3 of FCTC (tobacco industry interference in tobacco control activities) in India- a qualitative scoping study
Source: BMC Public Health. 2021 Oct 14;21:1855. doi: 10.1186/s12889-021-11773-x (PMC8515702; doi:10.1186/s12889-021-11773-x)
Supplement: Supplementary file 1 — Additional file 1. Supplementary File 1: In-depth interview Schedule. [file 12889_2021_11773_MOESM1_ESM.docx]

**Supplementary File 1: In-depth interview Schedule**

**Part 1: General Details:**

1. **Name :**
2. **Age :**
3. **Gender : Male / Female**
4. **Occupation Status : In service / Retired**
5. **Designation :**
6. **Organization : Government / Non-Government / Private**
7. **Address :**
8. **Contact Email :**
9. **Mobile Number :**

**Part 2: Interview Schedule:**

1. How long have/had you been associated with tobacco control activity and in what capacity?
   1. Can you briefly describe your activities in tobacco control (awareness generation/ enforcement of law/ implementation of program etc.)?
2. What are the common challenges you faced/ facing in the carrying out tobacco control activities?
   1. Why do you think that despite your best efforts these challenges come up?
3. In your opinion what constitutes tobacco industry? Can you name few of them?
4. Have you ever been approached by any tobacco industry personnel? How (face to face/representation/ third party/letters/telephonically/email etc.)
5. For what reasons/issues have you been approached by tobacco industry personnel?
6. Have you ever been offered any tangible (gifts/ sponsorships etc.) or intangible benefits (invitations/ recognitions etc.)?
7. Have you or your superiors/colleagues received threats from Tobacco Industry?
8. In your opinion are there any other informants who have been possibly been approached by tobacco industry personnel?
9. During the process of analysis if some more data is needed, can we contact you for it?

If case studies are developed from the current study, would you like to be approached/ contacted in future?
