# Supplementary material for: Evidence on article 5.3 of FCTC (tobacco industry interference in tobacco control activities) in India- a qualitative scoping study
Source: BMC Public Health. 2021 Oct 14;21:1855. doi: 10.1186/s12889-021-11773-x (PMC8515702; doi:10.1186/s12889-021-11773-x)
Supplement: Supplementary file 2 — Additional file 2. Supplementary File 2: Ranking Sheet: Perception of study participants regarding Tobacco Industry using ‘Power Ranking Methodology’. [file 12889_2021_11773_MOESM2_ESM.docx]

**Supplementary File - 2**

**Ranking Sheet: Perception of study participants regarding Tobacco Industry using ‘Power Ranking Methodology’.**

**Instruction:** Prioritize the below listed stakeholders as per your perception as representative of tobacco industry. Rank the stake holders from 1 to 17 such that 1 represents least representative and 17 correspond to most representative of tobacco industry.

| **SN** | **Stakeholders** | **Rank/Score** |
| --- | --- | --- |
|  | Wholesaler |  |
|  | Government without tobacco stocks |  |
|  | PR Company |  |
|  | Manufacturer |  |
|  | Vendors |  |
|  | Pension funds and other financial incentive schemes |  |
|  | Banks and financial institutions |  |
|  | Tobacco union workers |  |
|  | Government with tobacco stocks |  |
|  | Politicians |  |
|  | Advertisers |  |
|  | Farmers corporations |  |
|  | Bureaucrats |  |
|  | Bidi rollers |  |
|  | Farmers |  |
|  | Civil Society Organization |  |
|  | Hospitality Industry |  |
