# Supplementary material for: Evidence on article 5.3 of FCTC (tobacco industry interference in tobacco control activities) in India- a qualitative scoping study
Source: BMC Public Health. 2021 Oct 14;21:1855. doi: 10.1186/s12889-021-11773-x (PMC8515702; doi:10.1186/s12889-021-11773-x)
Supplement: Supplementary file 3 — Additional file 3. Supplementary File 3: Questionnaire: Perception of study participants regarding involvement of “Tobacco industry role players” in various identified themes. Instruction: Prioritize (1 to 17) the role players of tobacco industry under each theme of industry’s interferences. Rank the role players from 1 to 17 such that 1 represents least involvement and 17 correspond to most involvement under a particular theme of TII. [file 12889_2021_11773_MOESM3_ESM.docx]

**Supplementary File 3**

**Questionnaire: Perception of study participants regarding involvement of “Tobacco industry role players” in various identified themes.** *Instruction: Prioritize (1 to 17) the role players of tobacco industry under each theme of industry’s interferences. Rank the role players from 1 to 17 such that 1 represents least involvement and 17 correspond to most involvement under a particular theme of TII.*

| **S. N0** | **Tobacco industry role players** | **Theme 1** (*Influencing the policy and administrative decision*) | **Theme 2** (*Interference with implementation of tobacco control laws and activities*) | **Theme 3** (*False propaganda and hiding the truth*) | **Theme 4** (*Manipulating front action group*) | **Theme 5** (*Rampant TAPS activities*) | **Theme 6** *(Others)* |
| --- | --- | --- | --- | --- | --- | --- | --- |
|  | **Manufacturer** |  |  |  |  |  |  |
|  | **Wholesaler** |  |  |  |  |  |  |
|  | **Vendors** |  |  |  |  |  |  |
|  | **Advertisers** |  |  |  |  |  |  |
|  | **PR Company** |  |  |  |  |  |  |
|  | **Government with tobacco stocks** |  |  |  |  |  |  |
|  | **Government without tobacco stocks** |  |  |  |  |  |  |
|  | **Tobacco union workers** |  |  |  |  |  |  |
|  | **Farmers** |  |  |  |  |  |  |
|  | **Farmers corporations** |  |  |  |  |  |  |
|  | **Pension funds and other financial incentive schemes** |  |  |  |  |  |  |
|  | **Banks etc.** |  |  |  |  |  |  |
|  | **Bidi rollers** |  |  |  |  |  |  |
|  | **Politicians** |  |  |  |  |  |  |
|  | **Bureaucrats** |  |  |  |  |  |  |
|  | **Civil Society Organization** |  |  |  |  |  |  |
| **17** | **Hospitality Industry** | Y |  | Y | Y | Y | Y |
